# Identification and Splicing Characterization of Novel *TMC6* and *TMC8* Variants Associated With Epidermodysplasia Verruciformis in Three Chinese Families

**DOI:** 10.3389/fgene.2021.712275

**Published:** 2021-07-27

**Authors:** Rongrong Wang, Jiawei Liu, Xueting Yang, Xiaerbati Habulieti, Xue Yu, Liwei Sun, Han Zhang, Yang Sun, Donglai Ma, Xue Zhang

**Affiliations:** ^1^McKusick-Zhang Center for Genetic Medicine, State Key Laboratory of Medical Molecular Biology, Institute of Basic Medical Sciences Chinese Academy of Medical Sciences, School of Basic Medicine Peking Union Medical College, Beijing, China; ^2^Department of Dermatology, Peking Union Medical College Hospital, Chinese Academy of Medical Sciences, Beijing, China

**Keywords:** epidermodysplasia verruciformis, human beta HPV, *TMC6*, *TMC8*, pathogenic variants

## Abstract

**Background**: Epidermodysplasia verruciformis (EV) is a rare genodermatosis characterized by abnormal susceptibility to human beta papillomavirus infections and a particular propensity to develop non-melanoma skin cancers (NMSCs). The majority of EV cases are caused by biallelic null variants in *TMC6*, *TMC8*, and *CIB1*. This study aimed to identify disease-causing variants in three Chinese families with EV and to elucidate their molecular pathogenesis.

**Methods**: Genomic DNA from the probands of three EV families was analyzed by whole-exome sequencing (WES). cDNA sequencing was performed to investigate abnormal splicing of the variants. Quantitative RT-PCR (qRT-PCR) was conducted to quantify the mRNA expression of mutant *TMC6* and *TMC8*.

**Results**: Whole-exome sequencing identified two novel homozygous variants (c.2278-2A > G in *TMC6* and c.559G > A in *TMC8*) in families 1 and 2, respectively. In family 3, WES revealed a recurrent and a novel compound heterozygous variant, c.559G > A and c.1389G > A, in *TMC8*. The c.2278-2A > G *TMC6* variant led to the skipping of exon 19 and resulted in premature termination at codon 776. Subsequent qRT-PCR revealed that the aberrantly spliced transcript was partly degraded. Notably, the *TMC8* c.559G > A variant created a novel acceptor splice site at c.561 and yielded three different aberrant transcripts. qRT-PCR revealed that most of the mutant transcripts were degraded *via* nonsense-mediated mRNA decay (NMD).

**Conclusion**: We identified three novel disease-causing variants in *TMC6* or *TMC8* in three Chinese families with EV. The EV phenotypes of the three patients were due to a reduction in TMC6 or TMC8. Our findings expand the genetic causes of EV in the Chinese population.

## Introduction

Epidermodysplasia verruciformis (EV, MIM#226400) is a rare Mendelian genodermatosis originally described by Lewandowsky and Lutz (1922). EV is characterized by persistent, disseminated, refractory flat wart-like, and pityriasis versicolour-like lesions on the trunk, neck, arms, and face during childhood due to an abnormal susceptibility to specific human beta papillomavirus genotypes (including HPV-5, 8, 14, 20, etc.). Because of persistent EV-HPV infection, approximately half of EV patients develop non-melanoma skin cancers (NMSCs), mainly squamous cell carcinomas (SCCs), in sun-exposed areas before 40 years of age. In contrast, EV-HPV infection is non-pathogenic in the normal population (Orth, 2006; Przybyszewska et al., 2017). EV is generally inherited in an autosomal recessive manner, but X-linked recessive and autosomal dominant inheritance patterns have also been reported (Androphy et al., 1985; Orth, 2006; Przybyszewska et al., 2017). Biallelic null variants in *TMC6* (MIM: 605828) and *TMC8* (MIM: 605829) encoding EVER1 and EVER2 account for 50–60% of typical EV cases worldwide (Ramoz et al., 2002; Imahorn et al., 2017). Additionally, biallelic null variants in *CIB1* (MIM: 602293) encoding calcium- and integrin-binding protein-1 explain at least a portion of the remaining typical EV cases (de Jong et al., 2018). Recently, a limited number of patients with an EV-like phenotype have been described, namely, atypical EV. In contrast to typical EV patients, these patients not only suffer from EV-HPV-associated skin lesions but also co-infections with other viruses, bacteria, and fungi because of the T cell deficits caused by RHOH, IL7, MST1, CORO1A, TPP2, DCLRE1C, LCK, RASGRP1, and DOCK8 deficiencies (Crequer et al., 2012a,b; Stray-Pedersen et al., 2014; Horev et al., 2015; Stepensky et al., 2015; Li et al., 2016; Liu et al., 2017; Platt et al., 2017; Tahiat et al., 2017). More than 500 cases of typical EV have been described across different racial and ethnic groups, and several EV patients have been reported among the Chinese population; however, few of them have undergone detailed genetic analysis. Here, we report three novel pathogenic variants in *TMC6* and *TMC8* in three Chinese families with typical EV. Interestingly, we identified one recurrent *TMC8* missense variant that resulted in loss-of-function (LOF) of *TMC8 via* aberrant splicing and subsequent nonsense-mediated mRNA decay (NMD).

## Materials and Methods

### Ethics and Consent Statement

Three Chinese families were recruited in the present study. Clinical information and peripheral blood samples were collected from three probands and six unaffected individuals after obtaining individual written informed consent. This study was approved by the Institutional Review Board of Peking Union Medical College and was performed according to the Declaration of Helsinki.

### Whole-Exome Sequencing and Data Analysis

Genomic DNA was extracted from peripheral blood samples using a QIAamp DNA Blood Midi Kit (Qiagen, Hilden, Germany) according to the standard protocol. To identify the underlying genetic cause of EV in the three families, we performed whole-exome sequencing (WES) on the three probands. Briefly, genomic DNA was captured by the Agilent SureSelect^XT^ Human All Exon kit (Agilent Technologies, CA, United States) and sequenced on the Illumina HiSeq X platform (Illumina, CA, United States) with a 100 X read depth. Paired-end reads were aligned to the GRCh37/hg19 reference sequence using Burrows Wheeler Aligner software (BMA: version 0.7.8-r455). Variant calling was performed using SAMtools software (version 1.0). After variant detection, ANNOVAR was used for annotation. Then, variants were filtered based on the following criteria: (1) variants with a minor allele frequency (MAF) <1% were retained (the 1,000 Genomes Browser, dbSNP, gnomAD, and Exome Variant Server); (2) variants occurring in the coding region or splice sites were retained; and (3) homozygous or compound heterozygous variants were preferentially analyzed. All identified variants were further verified in available family members and 200 unrelated Chinese control individuals using Sanger sequencing. *In silico* prediction tools, such as SIFT, Polyphen2, REVEL, and InterVar, were used to predict the pathogenicity of the missense variants, while Human Splicing Finder 3.1 software (HSF) and NetGene2 were used to evaluate the effects of the variants on splicing.

### RNA Extraction, cDNA Synthesis, and Construct Construction

Total RNA was isolated from peripheral blood samples using TRIzol LS reagent (Invitrogen, CA, United States), and cDNA was obtained by reverse transcription using a PrimeScript™ RT reagent kit (TaKaRa, Dalian, China) according to the manufacturer’s protocol. To investigate the abnormal splicing of the c.2278-2A > G *TMC6* variant or the c.559G > A *TMC8* variant, we amplified *TMC6* or *TMC8* cDNA using primers in exons 16 and 20 of *TMC6* or exons 4 and 8 of *TMC8* (Supplementary Table S1) from the proband, the heterozygous carrier, and a control individual (C1). The obtained RT-PCR products were analyzed by gel electrophoresis on a 2% agarose gel and then cloned into the pMD18-T vector. In total, 50 positive recombinant plasmids underwent further Sanger sequencing.

### Quantitative RT-PCR

Quantitative RT-PCR (qRT-PCR) was carried out to quantify the mRNA expression of the mutant *TMC6* or *TMC8* in a QuantStudio 3 Real-Time PCR System (Applied Biosystems, MA, United States) using SYBR Premix Ex Taq (Takara, Dalian, China) with primer pairs for *TMC6* (E3-4F/E5R) or *TMC8* (E9F/E10R) according to the manufacturer’s protocol. Glyceraldehyde 3-phosphate dehydrogenase (GAPDH) was used as an endogenous control. The relative mRNA expression levels were calculated using the 2^−ΔΔ^Ct method. All reactions were run in triplicate or more, and data are presented as the means ± SD.

## Results

### Clinical Manifestations of the EV Families

**Figure 1:**
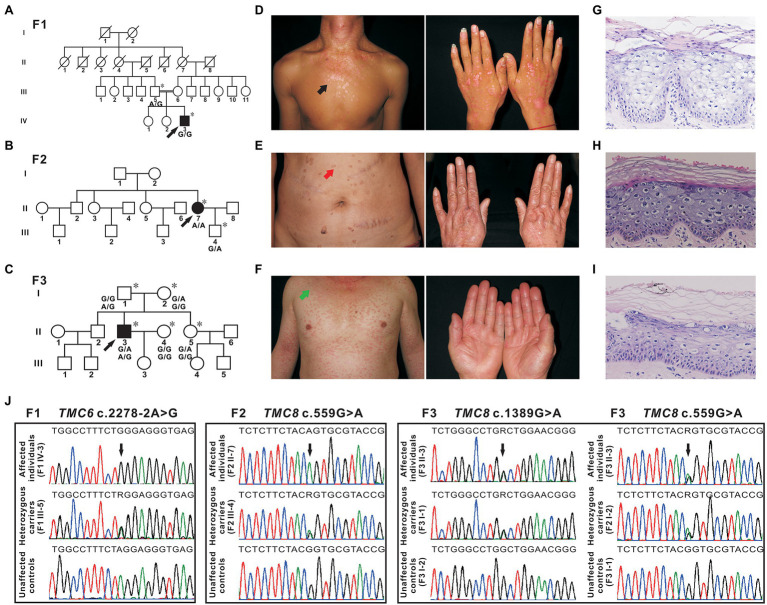
Pedigrees, clinical characteristics, and detection of variants in three Chinese epidermodysplasia verruciformis (EV) families. **(A–C)** Pedigrees of three family members affected by EV (F1, F2, and F3). The squares, circles, blackened, and open symbols indicate males, females, and affected and unaffected individuals, respectively. The arrows indicate the probands, and the asterisks denote the individuals available for genotyping. **(D)** Reddish papules coalesced into flat plaques on the neck and chest of patient IV-3 (F1, black arrow). **(E)** Verrucous hyperkeratotic brownish macules on the abdomen of patient II-7 (F2, red arrow). **(F)** Pityriasis versicolour-like lesions on the neck, trunk, and extremities of patient II-3 (F3, green arrow). **(G–I)** Histopathology of affected skin from three probands [patient IV-3 from F1 **(G)**, patient II-7 from F2 **(H)**, and patient II-3 from F3 **(I)**], hyper/parakeratosis, and large numbers of nests of koilocytes in the granular and spinous layers with a clear, light-blue pale cytoplasm. H&E (×200). **(J)** Sequencing chromatograms of affected individuals, heterozygous carriers of *TMC6*, or *TMC8* variants. Unaffected controls are also shown. The black arrows indicate the sites of the variants, and R (a degenerate base) indicates **A** and **G**.

The proband from family 1 (Figure 1A, IV-3) was a 23-year-old male. The lesions first presented as erythaematous keratotic papules and plaques on the back of his hands 17 years ago and then gradually spread over his whole body (Figure 1D). His parents were first cousins. He did not suffer from other infections or NMSC. Oncogenic HPV-14 was detected in skin biopsy tissues by DNA sequencing. The proband from family 2 (Figure 1B, II-7) was a 51-year-old woman who presented with a 40-year history of multiple verrucous hyperkeratotic brownish macules on her face, trunk, and hands (Figure 1E). The proband’s parents denied consanguinity between their families, and there was no family history of other infections or NMSC. HPV typing was performed on DNA isolated from skin lesions, and the HPV genotype identified was HPV-5. The proband from family 3 (Figure 1C, II-3), a 46-year-old man, presented with disseminated pityriasis versicolour-like lesions on sun-exposed areas at 17 years of age. The skin lesions extended to his chest, extremities, abdomen, and back with aging (Figure 1F). Apart from these dermatologic signs, he was otherwise healthy and developed normally. The skin lesions on sun-exposed areas developed into basal cell carcinoma repeatedly and were excised by conventional surgery. Infection with HPV-5 was confirmed by DNA sequencing. Skin biopsy of the lesions from the patients showed enlarged nests of koilocytes with a clear, pale-stained cytoplasm in the granular and spinous layers and hyper/parakeratosis (Figures 1G–I). From these observations, the diagnosis of EV was made.

### Genomic Sequencing and *in silico* Analysis

**Figure 2:**
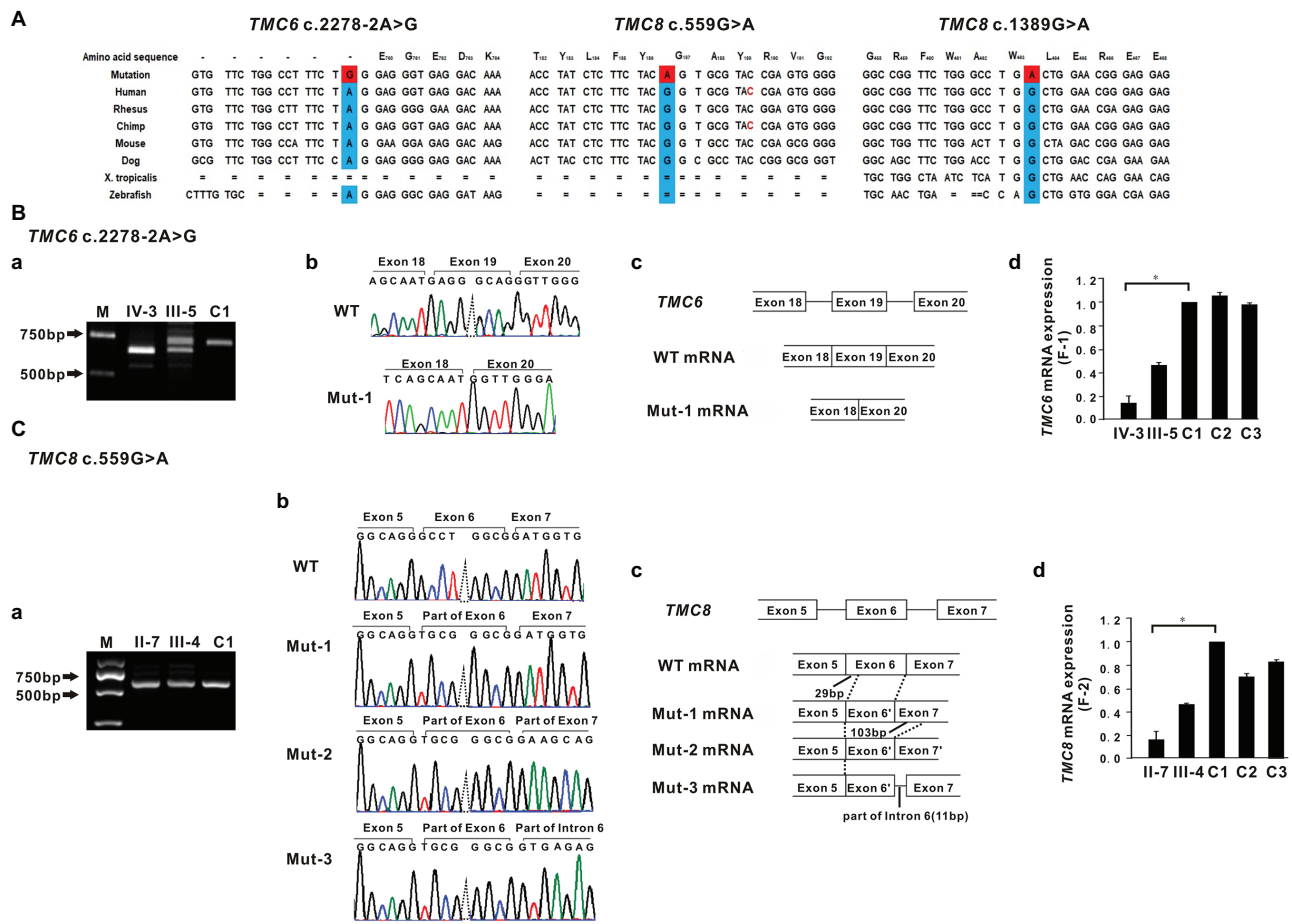
Sequence conservation and functional analysis of the identified *TMC6* and *TMC8* variants. **(A)** All three variants, c.2278-2A>G in *TMC6*, c.559G>A in *TMC8*, and c.1389G>A in *TMC8*, detected in the three affected individuals are evolutionarily conserved across different species. **(B-a)** RT-PCR of mRNA from peripheral blood lymphocytes from patient IV-3 (F1) yielded a shorter band than that of the control (C1), indicating an aberrant transcript. M, marker. **(B-b)** Sequencing traces of the RT-PCR products showing that the wild-type (WT, top) and *TMC6* exon 19 skipped transcripts (Mut-1, bottom). **(B-c)** Schematic representation of exon 18 to exon 20 of *TMC6* showing normal or aberrant splicing. The boxed regions denote exons, while the connecting solid lines indicate introns. **(B-d)** Quantitative RT-PCR (qRT-PCR) of the relative *TMC6* mRNA expression in peripheral blood lymphocytes from the affected individual (IV), the heterozygous carrier (III-5), and the independent controls (C1, C2, and C3; right). **(C–a)** Bands approximately 650 bp in size were observed in both the proband II-7 and C1. **(C-b)** Sequencing chromatograms showed that the *TMC8* c.559G > A variant had a novel acceptor splice site at c.561 and yielded three different aberrant transcripts (Mut-1, Mut-2, and Mut-3). **(C-c)** Schematic representation of the critical region of *TMC8* showing normal or aberrant splicing. The solid black lines indicate the location and number of inserted or deleted nucleotides. **(C-d)** qRT-PCR of the relative *TMC8* mRNA expression in peripheral blood lymphocytes from the affected individual (II-7), the heterozygous carrier (III-4), and the independent controls (C1, C2, and C3; right). For all qRT-PCR assays, glyceraldehyde 3-phosphate dehydrogenase (GAPDH) was used as an endogenous control. C1 was set to 1.0, and data are presented as the mean ± SD (*n* ≥ 3, ^*^p < 0.05).

In family 1, a novel homozygous splice site variant in the acceptor splice site of intron 18 of *TMC6* (c.2278-2A > G, NM_007267.7) was identified in the proband. His father was a heterozygous carrier of the identified variant (Figure 1J). This variant was not found in 200 unrelated Chinese controls or in public databases. Using HSF and NetGene2, *in silico* analyses revealed that the variant destroyed the acceptor site, which most likely affected splicing. In family 2, a novel homozygous missense variant, c.559G > A (p. Gly187Ser), in exon 6 of *TMC8* (NM_152468.4) was found in the patient, whereas her son was heterozygous for this variant (Figure 1J). The c.559G > A variant was found to be present in two individuals in gnomAD (MAF = 0.00000941), but was not found in 200 unrelated Chinese controls. The variant was classified as probably damaging or damaging using *in silico* analysis tools, such as SIFT, Polyphen2, and REVEL, and was categorized as having uncertain significance in InterVar. In addition, analysis of c.559G > A with HSF showed no significant splicing motif alterations, while NetGene2 revealed that the c.559G > A variant may create a novel acceptor splice site at c.561 (confidence: 0.97). In family 3, a recurrent and a novel compound heterozygous variant, c.559G > A and c.1389G > A (p. Trp463*), in *TMC8* were found in the proband, and these two variants co-segregated with the EV phenotype within the family (Figures 1C,J). The c.1389G > A nonsense variant was not found in 200 unrelated Chinese controls or in public databases. HSF and NetGene2 analysis revealed that the c.1389G > A variant had no significant impact on splicing signals; therefore, it may lead to either synthesis of truncated protein products lacking the key TMC domain or degradation *via* NMD. All three variants are evolutionarily conserved across different species (Figure 2A). No pathogenic variants in other known EV genes, such as *CIB1*, *RHOH*, *IL7*, *MST1*, *CORO1A*, *TPP2*, *DCLRE1C*, *LCK*, *RASGRP1*, and *DOCK8*, were identified in any of the three probands.

### mRNA Expression of Mutant *TMC6*

To investigate the impact of the c.2278-2A > G variant on the splicing of *TMC6* mRNA, we amplified *TMC6* cDNA using primers in exons 16 and 20 from proband IV-3 of F1, heterozygous carrier III-5, and a control individual (C1). As shown in Figure 2B–a, a smaller RT-PCR product was observed *via* agarose gel electrophoresis in the proband compared to the expected DNA band observed in C1, while two DNA bands were detected in the heterozygous carrier. Sequencing confirmed that the shorter RT-PCR products corresponded to an abnormal mRNA transcript with complete skipping of exon 19 of *TMC6* (r.2278_2354del, Figures 2B–b,B–c). This deletion disrupted the open reading frame and resulted in premature termination at codon 776 (p. Glu760Glyfs*17). Subsequent qRT-PCR revealed that peripheral blood lymphocytes from proband IV-3 had much lower levels of *TMC6* mRNA than those from the controls (C1, C2, and C3), whereas the heterozygous carrier had intermediate RNA levels, suggesting that the aberrantly spliced transcript was partly degraded (Figure 2B–d).

### mRNA Expression of Mutant *TMC8*

Since the currently known *TMC8* variants are all truncated variants, we further evaluated the effect of the c.559G > A missense variant on the expression of *TMC8* mRNA. Interestingly, quantification of *TMC8* mRNA showed a significant reduction of the transcript in peripheral blood lymphocytes from proband II-7 of F2 compared to that in blood lymphocytes from the independent controls (Figure 2C–d). To explore the reasons for the decrease in mutant *TMC8* mRNA, the targeted fragments from the patients’ cDNA were amplified using primers in exons 4 and 8 of *TMC8*. Bands approximately 650 bp in size were observed in both the proband and C1 (Figure 2C–a). Then, the obtained RT-PCR products were cloned into the pMD18-T vector for sequencing. cDNA analysis revealed that the *TMC8* c.559G > A variant directly created a novel acceptor splice site at c.561 and yielded three different aberrant transcripts (Figures 2C–b,C–c), of which the mut-1 isoform accounted for more than 70% of all transcripts. Mut-1 yields a 255-amino acid truncated protein, while the other two aberrant transcripts caused a frameshift at amino acid position 178 of TMC8 and led to a 682-amino acid or a 720-amino acid truncated protein, respectively. Our results suggested that the c.559G > A variant, which introduced a premature termination codon (PTC), might lead to the degradation of most of the mutant transcripts *via* NMD.

## Discussion

**Figure 3:**
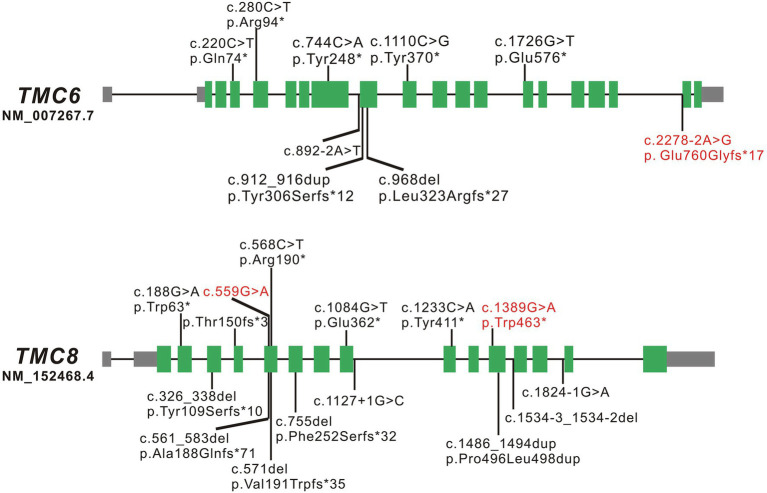
Schematic diagrams of *TMC6* and *TMC8*. The green boxed regions denote exons, gray boxed regions represent 5'- or 3'-untranslated regions, and connecting solid lines indicate introns. The variants marked in red are the novel variants detected in the present study; those marked in black are the variants reported to date.

*TMC6* and *TMC8* are two adjacent and related genes located on chromosome 17q25.3. *TMC6* is approximately 19.51 kb and consists of 20 constitutive exons, while *TMC8* is approximately 12.198 kb and comprises 16 exons. *TMC6* and *TMC8* encode transmembrane channel-like proteins of 805 amino acids and 726 amino acids, respectively that have a functionally important, conserved 120-amino acid domain known as the TMC domain. Both TMC6 and TMC8 proteins are widely expressed throughout the body, and they are mainly located in the endoplasmic reticulum (Orth, 2006). To date, eight LOF variants of *TMC6* and 13 LOF variants of *TMC8*, located in different exons across the genes, have been reported, which are summarized in Figure 3 and Supplementary Table S2. There have been several clinical reports on EV patients in China; however, few of them have undergone detailed genetic analysis. Sun et al. (2005) identified a homozygous c.568C > T (p. Arg190*) variant in *TMC8* in a Chinese patient born from a first-cousin marriage. Subsequently, Zuo et al. (2006) detected a homozygous small insertion (c.912_916dupCATGT; p. Tyr306Serfs*12) in another consanguineous Chinese family with EV. Here, to the best of our knowledge, we report three previously unreported variants in *TMC6* and *TMC8* in three unrelated patients with different skin manifestations. Interestingly, the *TMC8* variant (c.559G > A), ostensibly designated a missense variant, generated a new acceptor splice site at c.561 that was recognized by the splicing machinery. Sanger sequencing showed that all 50 tested transcripts were aberrant transcripts using the new acceptor splice site, and no normal splicing mRNA was detected in our analysis. qRT-PCR revealed that although most of the abnormal mRNA transcripts of *TMC8* with PTC were degraded, presumably *via* NMD, approximately 15% of mRNA was retained, suggesting a low level of truncated TMC8 protein in the patient. In addition to this, the *TMC8* variant showed deleterious prediction scores from different *in silico* tools, and the online tool I-Mutant v2.0 suggested that the TMC8 p. Gly187Ser protein had obviously decreased stability, with a predicted DDG of −3.04 (DDG<0: decrease stability). Therefore, we speculated that it might play a role in the pathogenesis of EV as missense variant even if this variant had no potential effect on pre-mRNA splicing. However, further investigation is needed. It has been reported that most previously described null variants in *TMC6* or *TMC8* introduce PTCs and presumably destabilize mutant transcripts by NMD, resulting in a reduction of the TMC6 or TMC8 proteins (Ramoz et al., 2002; Gober et al., 2007; Miyauchi et al., 2016). Nevertheless, recently, it has been reported that splice-site variants of *TMC8* may cause LOF of TMC8 due to the partial loss of the TMC domain (Miyauchi et al., 2016; Imahorn et al., 2017). According to our results, all three variants were classified as pathogenic according to the ACMG criteria (Richards et al., 2015). We speculated that the EV phenotypes of the three patients with variants in *TMC6* or *TMC8* in the present study were mainly due to a reduction in TMC6 or TMC8. However, the mechanism by which LOF in *TMC6* or *TMC8* can cause continuous symptomatic infections with HPV in EV patients needs to be further investigated.

Subsequently, we analyzed the clinical characteristics of the cases in the present study and published cases associated with *TMC6* or *TMC8* variants. Due to the incomplete clinical information of many EV-affected family members, we only focused on the clinical characteristics of the probands. As shown in Supplementary Table S2, the age of onset of EV in these patients varied from 4 to 20 years old (median: 10), and the male/female ratio was 2.1:1. Sixty-four percent of cases with EV (16/25) originated from consanguineous families, and 48% (12/25) of EV patients developed NMSC (such as SCC and/or basal cell carcinoma) before 61 years of age, of which the youngest patient suffered from BCC at 22 years of age. One patient with *TMC8* variants suffered from Merkel cell carcinoma at 82 years of age (Mizuno et al., 2015). Consistent with the previous report by Imahorn et al. (2017), the most frequent EV-HPV in the 25 patients was HPV-5 (12/25), followed by HPV-14 (7/25) and HPV-20 (4/25), and the rare EV-HPVs included HPV-17, 22, 38, 93, 3, 8, 9, 12, 21–24, 47, and so on. Interestingly, except for two EV patients (one of the patients was only 18 years old), most of the EV patients with potentially oncogenic HPV-5 infection developed NMSC.

In summary, our finding of three novel variants in either *TMC6* or *TMC8* expands the genetic causes of EV in the Chinese population. Notably, an increasing number of examples have revealed that exonic mutations (both missense and synonymous mutations) disrupt the binding of splicing factors to these sequences or generate new splice sites or regulatory elements, causing disease (Claverie-Martin et al., 2015). Combining our findings, the non-truncating variants found in *TMC6* or *TMC8* are worthy of further investigation of their genetic mechanisms.

## Data Availability Statement

The datasets for this article are not publicly available due to concerns regarding participant/patient anonymity. Requests to access the datasets should be directed to the corresponding authors.

## Ethics Statement

The studies involving human participants were reviewed and approved by the Institutional Review Board of Peking Union Medical College. The patients/participants provided their written informed consent to participate in this study. Written informed consent was obtained from the individual(s) for the publication of any potentially identifiable images or data included in this article.

## Author Contributions

RW, JL, DM, and XZ contributed to the conception and design of the study. RW, XYa, XH, XYu, LS, HZ, and YS contributed to the acquisition and analysis of the data. RW, JL, XYa, and XH contributed to the drafting of the manuscript and figures. All authors agree to be accountable for the content of the work and contributed to the article and approved the submitted version.

## Conflict of Interest

The authors declare that the research was conducted in the absence of any commercial or financial relationships that could be construed as a potential conflict of interest.

## Publisher’s Note

All claims expressed in this article are solely those of the authors and do not necessarily represent those of their affiliated organizations, or those of the publisher, the editors and the reviewers. Any product that may be evaluated in this article, or claim that may be made by its manufacturer, is not guaranteed or endorsed by the publisher.
