# Supplementary material for: Identification and Splicing Characterization of Novel TMC6 and TMC8 Variants Associated With Epidermodysplasia Verruciformis in Three Chinese Families
Source: Front Genet. 2021 Jul 27;12:712275. doi: 10.3389/fgene.2021.712275 (PMC8353250; doi:10.3389/fgene.2021.712275)
Supplement: Supplementary file 1 [file Data_Sheet_1.docx]

Supplementary Material

**Supplementary Table S1 List of primers used in this study**

| Primer Name | Forward Primer (5’-3’) | Reverse Primer (5’-3’) |
| --- | --- | --- |
| Primer sequences for cDNA sequencing and splicing analysis | | |
| *TMC6*(E16F/ E20R) | CACACATGAGCACCGTCTTC | TAAATAGAGTCCCAGGCAGG |
| *TMC8*(E4F/ E8R) | ACTTCACCTTCCTCCGCTTC | AGCCCGTTGAGTACGTTGAC |
| Primer sequences for qRT-PCR analysis | | |
| *TMC6* (E3-4F/ E5R) | TGACAGGAAGTAGCCAGCAG | TACTGGGAGATGATGGCACC |
| *TMC8* (E9F/ E10R) | ACTACCCTCCCAACACGGAG | TGTCTCTGCCAATGCACAGT |
| *GAPDH* | tgcaccaccaactgcttag | caggcagggatgatgttc |

**Supplementary Table S2 Summary of the clinical features of the cases included in the present study and published cases associated with *TMC6* or *TMC8* variants**

| **No.** | **Age** | **Gender** | **Age of onset** | **Clinical features** | **No. of patients in the**  **families** | **Extracutaneous manifestations** | **HPV genotypes** | **Skin cancer/Age/Location** | **Consanguinity** | **Gene** | **Variants** | **Origin** | **References** |
| --- | --- | --- | --- | --- | --- | --- | --- | --- | --- | --- | --- | --- | --- |
| **P1^a^** | 37 | M | 6 | Flat wart-like lesions, pityriasis versicolour-like lesions on the face, neck, and limbs | 2 | None | HPV-5,14,20 | BCC/NA/NA | Yes | *TMC6* | c.280C>T, p. Arg94* | Algeria | Ramoz et al., 2002; Ramoz et al., 1999 |
| **P2** | 37 | F | 10 | Flat wart-like lesions, pityriasis versicolour-like lesions on the face, neck, and limbs | 1 | None | HPV-5,14,20 | NA | Yes | *TMC6* | c.280C>T, p. Arg94* | Algeria | Ramoz et al., 2002; Ramoz et al., 1999 |
| **P3^a^** | 38 | M | 6 | Flat wart-like lesions, pityriasis versicolour-like lesions all over the body | 3 | None | HPV-5,17 | NA | Yes | *TMC6* | c.1726G>T,  p. Glu576* | Colombia | Ramoz et al., 2002; Ramoz et al., 1999 |
| **P4^a^** | 65 | F | Teenager | Flat warts on the face and hands | 3 | Gastric cancer | NA | SCC,  Bowen disease  /51,52,54/hand;face | No | *TMC6* | c.744C>A,  p. Tyr248*;  c.892-2A>T | Japan | Tate et al., 2004 |
| **P5^a^** | 23 | M | 18 | Flat-wart-like papules, sporadic brownish pityriasis versicolour-like lesions on his face, arms, and the trunk | 2 | None | NA | None | Yes | *TMC6* | c.912_916  dup,  p.Tyr306Serfs*12 | China | Zuo et al., 2006 |
| **P6** | 57 | F | 11 | Scaly erythaematous plaques on the neck, trunk and extremities | 1 | None | HPV-12 | None | Yes | *TMC6* | c.220C>T,  p. Gln74* | Japan | Aochi et al., 2007 |
| **P7** | 34 | M | 14 | Multiple verrucous lesions on his face, torso, and extremities | 1 | None | HPV-17,DL473, SK3, GRT04 | None | Yes | *TMC6* | c.968del,  p. Leu323Argfs*27 | Pakistan | Gober et al., 2007 |
| **P8^a^** | 52 | F | 10 | Flat warts on the upper limbs and trunk, few papules in hands | 2 | Hepatitis C infection | HPV-5,22 | Bowenoid, SCC/47/Forehead | NA | *TMC6* | c.1110C>G, p. Tyr370* | Frence | Youssefian et al., 2019 |
| **P9^a^** | 44 | F | 7 | Warty, non-pruritic, hyperkeratotic, mostly reddish-brown skin lesions on the scalp, neck, chest, back, arms and thighs | 6 | None | NA | SCC/NA/NA | Yes | *TMC6* | c.1110C>G, p. Tyr370* | Mexico | López-Ramírez et al., 2020 |
| **P10** | 23 | M | 6 | Erythaematous keratotic papules and plaques scattered over the whole body | 1 | None | HPV-14 | None | Yes | *TMC6* | c.2278-2A>G | China | The present study |
| **P11^a^** | 32 | M | 7-10 | Flat wart-like lesions on the face and the dorsa of the hands, pityriasis versicolour-like lesions on the trunk | 5 | Severely mentally retarded | HPV-38, 3 | None | Yes | *TMC8* | c.1084G>T,  p. Glu362* | Colombia | Ramoz et al., 2002; Ramoz et al., 2000 |
| **P12^a^** | 36 | F | NA | Flat warts, pityriasis versicolour‐like lesions, actinic keratoses | 3 | None | HPV-5b,20, 14d | Bowen's disease; SCC/NA/back, upper leg | Yes | *TMC8* | c.188G>A, p. Trp63* | Brazil | Rady et al., 2007; de Oliveira et al., 2004 |
| **P13** | 28 | M | 8 | Persistent flat warts and pityriasis versicolour-like lesions disseminated on the face, neck, and limbs | 1 | None | HPV-5 | BCC/26/forehead;  SCC/26/left temple | Yes | *TMC8* | c. 755del,  p.Phe252Serfs*32 | Algeria | Ramoz et al., 2002; |
| **P14** | 60 | M | Childhood | Whitish pityriasis versicolour-like lesions on the back | 1 | None | HPV-5, 14,21,93,96,20 | SCC, BCC/42/medial left eyelid | Yes | *TMC8* | c.326_338del,  p.Tyr109Serfs*10 | Italy | Landini et al., 2012 |
| **P15** | 58 | M | NA | Erythaematous and hyperkeratotic plaques | NA | None | HPV-5, 14,93,  24 | Bowen's disease/42/  face, back | NA | *TMC8* | c.571del,  p.Val191Trpfs*35 | Italy | Landini et al., 2012 |
| **P16** | 24 | M | 7 | Flat warts mainly on the face, arms and hands. pityriasis versicolour with slightly scaly macules on the neck and trunk | 1 | Hepatitis B infection | NA | None | Yes | *TMC8* | c.568C>T, p. Arg190* | China | Sun et al., 2005 |
| **P17** | 22 | M | 12 | Flat warts on the face, chest, and extremities | 1 | None | HPV-5, 14d,23, 38 | SCC/22/chest wall, cheek, left lateral canthus, and right preauricular;BCC/22/scalp | No | *TMC8* | c.561_583del,  p.Ala188Glnfs*71 | Hispanics | Berthelot et al., 2007 |
| **P18** | NA | NA | NA | NA | NA | NA | NA | NA | NA | *TMC8* | p.Thr150fs*3 | Poland | Lazarczyk et al., 2008 |
| **P19** | 82 | M | Twenties | Flat verruous papules and brown macules on the neck, face and chest | 1 | chronic obstructive pulmonary disease; bacterial pneumonia | HPV-16 | Bowen’s disease, BCC, and SCC/57/scalp, face, and neck; MCC/82/left cheek | NA | *TMC8* | c.1824-1G>A | Japan | Mizuno et al., 2015 |
| **P20** | 60 | M | Early childhood | Multiple brownish pityriasis versicolour-like macules and seborrhoeic keratosis-like nodules scattered mainly on sun-exposed areas | 1 | None | HPV-8, 22, 47 | None | Yes | *TMC8* | c.1534‐3_1534‐2delCA, p.Tyr512_Gln535del;p.Tyr512_Gln535del and p. Tyr 572_Ser608delinsCys | Japan | Miyauchi et al., 2016 |
| **P21^a^** | 18 | NA | Early childhood | Plane warts on chest and hands | 6 | None | HPV-5, 9 | None | Yes | *TMC8* | c.1127+1G>C | Turkey | Imahorn et al., 2017 |
| **P22** | >61 | M | 4 | Flat warts in hands and feet, tinea versicolour-like lesions on the face and neck | 1 | None | NA | SCC, basosquamous Carcinoma/61/face | NA | *TMC8* | c.1486_1494dup,  p.Pro496_Leu498dup | NA | Youssefian et al., 2019 |
| **P23** | NA | NA | 14 | Flat warts in hands and feet, tinea versicolour-like lesions on the face and neck | 1 | None | NA | None | Yes | *TMC8* | c.1233C>A, p. Tyr411* | NA | Youssefian et al., 2019 |
| **P24** | 51 | F | 11 | Multiple verrucous hyperkeratotic brownish macules on her face, trunk, and hands | 1 | None | HPV-5 | None | No | *TMC8* | c.559G>A | China | The present study |
| **P25** | 46 | M | 17 | Disseminated pityriasis versicolour-like lesions on the chest, extremities, abdomen, and back | 1 | None | HPV-5 | BCC/NA/on the sun-exposed areas | No | *TMC8* | c.559G>A; c.1389G>A, p. Trp463* | China | The present study |

P, patient; M, male; F, female; NA, not applicable; SCC, squamous cell carcinoma; BCC, [basal cell carcinoma](http://www.baidu.com/link?url=4eTvB_Oxao_rDcTGYX30uSl78xbE8PPD0p_byV7S1Q3booObf29825I8v5Ov53WDUFpmn4a3mi72HS-5aLDRVo4Pgnm_wzEn0ZDYlx2Bp62CEVID9HVZIy3VMymVJLYG); MCC, Merkel cell carcinoma; ^a^ only the clinical features of the probands are listed in Table 1.

**Supplementary Reference**

Aochi, S., Nakanishi, G., Suzuki, N., Setsu, N., Suzuki, D., Aya, K., et al. (2007). A novel homozygous mutation of the EVER1/TMC6 gene in a Japanese patient with epidermodysplasia verruciformis. Br. J. Dermatol. 157, 1265-1266. doi: 10.1111/j.1365-2133.2007.08206.x.

Berthelot, C., Dickerson, M.C., Rady, P., He, Q., Niroomand, F., Tyring, S.K., et al. (2007). Treatment of a patient with epidermodysplasia verruciformis carrying a novel EVER2 mutation with imiquimod. J. Am. Acad. Dermatol. 56, 882-886. doi: 10.1016/j.jaad.2007.01.036.

de Oliveira, W.R., He, Q., Rady, P.L., Hughes, T.K., Neto, C.F., Rivitti, E.A., et al. (2004). HPV typing in Brazilian patients with epidermodysplasia verruciformis: high prevalence of EV-HPV 25. J. Cutan. Med. Surg. 8, 110-115. doi: 10.1007/s10227-003-0100-6.

Gober, M.D., Rady, P.L., He, Q., Tucker, S.B., Tyring, S.K., Gaspari, A.A. (2007). Novel homozygous frameshift mutation of EVER1 gene in an epidermodysplasia verruciformis patient. J. Invest. Dermatol. 127, 817-820. doi: 10.1038/sj.jid.5700641.

Imahorn, E., Yüksel, Z., Spoerri, I., Gürel, G., Imhof, C., Saraçoğlu, Z.N., et al. (2017). Novel TMC8 splice site mutation in epidermodysplasia verruciformis and review of HPV infections in patients with the disease. J. Eur. Acad. Dermatol. Venereol. 31, 1722-1726. doi: 10.1111/jdv.14431.

Landini, M.M., Zavattaro, E., Borgogna, C., Azzimonti, B., De Andrea, M., Colombo, E., et al. (2012). Lack of EVER2 protein in two epidermodysplasia verruciformis patients with skin cancer presenting previously unreported homozygous genetic deletions in the EVER2 gene. J. Invest. Dermatol. 132, 1305-1308. doi: 10.1038/jid.2011.399.

Lazarczyk, M., Pons, C., Mendoza, J.A., Cassonnet, P., Jacob, Y., Favre M. (2008).Regulation of cellular zinc balance as a potential mechanism of EVER-mediated protection against pathogenesis by cutaneous oncogenic human papillomaviruses. J. Exp. Med. 205,35-42. doi: 10.1084/jem.20071311.

López-Ramírez, S., Santillán-Hernández, Y., Carrasco-Gerard, E., Rodas-Serrano, A., Zenteno, J.C. (2020). Next-Generation Sequencing Identifies a Homozygous Nonsense p.Tyr370* Mutation of the TMC6 Gene in a Mexican Pedigree with Epidermodysplasia Verruciformis. Rev. Invest. Clin. doi: 10.24875/RIC.20000415.

Miyauchi, T., Nomura, T., Suzuki, S., Takeda, M., Shinkuma, S., Arita, K., et al. (2016). Genetic analysis of a novel splice-site mutation in TMC8 reveals the in vivo importance of the transmembrane channel-like domain of TMC8. Br. J. Dermatol. 175, 803-806. doi: 10.1111/bjd.14569.

Mizuno, Y., Kato, G., Shu, E., Ohnishi, H., Fukao, T., Ohara, O., et al. (2015). Merkel cell polyomavirus-positive Merkel cell carcinoma in a patient with epidermodysplasia verruciformis. Acta. Derm. Venereol. 95:98-99. doi: 10.2340/00015555-1868.

Rady, P.L., De Oliveira, W.R., He, Q., Festa, C., Rivitti, E.A., Tucker, S.B., et al. (2007). Novel homozygous nonsense TMC8 mutation detected in patients with epidermodysplasia verruciformis from a Brazilian family. Br. J. Dermatol. 157, 831-833. doi: 10.1111/j.1365-2133.2007.08123.x.

Ramoz, N., Rueda, L.A., Bouadjar, B., Favre, M., Orth, G. (1999). A susceptibility locus for epidermodysplasia verruciformis, an abnormal predisposition to infection with the oncogenic human papillomavirus type 5, maps to chromosome 17qter in a region containing a psoriasis locus. J. Invest. Dermatol. 112, 259-263. doi: 10.1046/j.1523-1747.1999.00536.x.

Ramoz, N., Rueda, L.A., Bouadjar, B., Montoya, L.S., Orth, G., Favre, M. (2002). Mutations in two adjacent novel genes are associated with epidermodysplasia verruciformis. Nat. Genet. 32, 579-581. doi: 10.1038/ng1044.

Ramoz, N., Taïeb, A., Rueda, L.A., Montoya, L.S., Bouadjar, B., Favre, M., et al. (2000). Evidence for a nonallelic heterogeneity of epidermodysplasia verruciformis with two susceptibility loci mapped to chromosome regions 2p21-p24 and 17q25. J. Invest. Dermatol. 114, 1148-1153. doi: 10.1046/j.1523-1747.2000.00996.x.

Sun, X.K., Chen, J.F., Xu, A.E. (2005). A homozygous nonsense mutation in the EVER2 gene leads to epidermodysplasia verruciformis. Clin. Exp. Dermatol. 30, 573-574. doi: 10.1111/j.1365-2230.2005.01858.x.

Tate, G., Suzuki, T., Kishimoto, K., Mitsuya, T. (2004). Novel mutations of EVER1/TMC6 gene in a Japanese patient with epidermodysplasia verruciformis. J. Hum. Genet. 49, 223-225. doi: 10.1007/s10038-004-0135-6.

Youssefian, L., Vahidnezhad, H., Mahmoudi, H., Saeidian, A.H., Daneshpazhooh, M., Kamyab Hesari, K. et al. (2019) Epidermodysplasia Verruciformis: Genetic Heterogeneity and EVER1 and EVER2 Mutations Revealed by Genome-Wide Analysis. J. Invest. Dermatol. 139, 241-244. doi: 10.1016/j.jid.2018.07.010.

Zuo, Y.G., Ma, D., Zhang, Y., Qiao, J., Wang B. (2006). Identification of a novel mutation and a genetic polymorphism of EVER1 gene in two families with epidermodysplasia verruciformis. J. Dermatol. Sci. 44, 153-159. doi: 10.1016/j.jdermsci.2006.08.013.
